# The effect of vitamin D supplementation on some metabolic parameters in patients with nonalcoholic fatty liver disease: A systematic review and meta-analysis of 8 RCTs

**DOI:** 10.1097/MD.0000000000035717

**Published:** 2023-10-20

**Authors:** Xuemeng Chen, Ye Zhao, Ran Zhang, Yan Zhao, Liheng Dai

**Affiliations:** a Department of Public Health, International College, Krirk University, Bangkok, Thailand; b School of Architecture & Art Design, Hebei University of Technology, Tianjin, China; c Department of Internal Medicine, Tianjin Beichen Traditional Chinese Medicine Hospital, Tianjin, China.

**Keywords:** 25(OH)D, meta-analysis, nonalcoholic fatty liver, randomized controlled trial, vitamin D

## Abstract

**Background:**

To systematically evaluate the effects of vitamin D supplementation in patients with nonalcoholic fatty liver disease (NAFLD).

**Methods:**

National Library of Medicine, Cochrane Library, Elsevier, China National Knowledge Infrastructure, Web of Science, WANFANG databases, and Google Scholar were retrieved to collect relevant randomized controlled trials, which are published from the earliest records the time the database was created to April 2023. Meta-analysis was conducted by using Review Manager 5.4 software after evaluating in terms of inclusion and exclusion criteria. The outcome indicators include 25-hydroxyvitamin D [25(OH)D] levels, insulin resistance index (homeostasis model assessment of insulin resistance), fasting blood glucose, and fasting insulin levels (FINS).

**Results:**

Eight randomized controlled trials with a total of 657 patients are included. Vitamin D supplementation increased 25(OH)D levels significantly (mean difference [MD] = 2.01, 95% confidence intervals [CI]: 0.94 to 3.08, *P* < .05) and vitamin D supplementation had a significant effect on insulin resistance index (MD = −0.54, 95% CI: −1.28 to 0.20, *P* = .16), fasting glucose (MD = −0.59, 95% CI: −1.50 to 0.32, *P* = .20), and FINS levels (MD = −0.30, 95% CI: −0.77 to 0.17, *P* = .21) had no significant effect.

**Conclusion:**

Vitamin D supplementation improves 25(OH)D levels in patients with nonalcoholic fatty liver disease, but there is no effect on homeostasis model assessment of insulin resistance, fasting blood glucose, or FINS.

## 1. Introduction

Vitamin D is fat-soluble. It has effects in type 2 diabetes mellitus, metabolic syndrome, cardiovascular disease, cancer, and autoimmune diseases.^[1]^ Nonalcoholic fatty liver disease (NAFLD) is a metabolic disease of liver.^[2]^ The incidence of NAFLD increases in the whole world.^[3]^ Lifestyle interventions, dietary restrictions and physical exercise are recommended; however, the effects are usually limited and short-lived.^[4]^ It shows that insulin resistance (IR), oxidative stress, and impaired glucolipid metabolism play a key role in the development of NAFLD; patient with NAFLD has a high risk of developing type 2 diabetes mellitus or metabolic syndrome.^[5]^ Insulin resistance is the biggest contributor to this condition.^[6]^ It shows that a reduced vitamin D level is strongly associated with the pathogenesis of IR, so vitamin D supplementation may have a significant improvement in glycemic control.^[7]^ In pancreatic β-cells, 25-hydroxyvitamin D [25(OH)D] binds to the vitamin D receptor, which stimulates the insulin secretion.^[8]^ Vitamin D in patients with NAFLD has controversial findings. This study intends to conduct a systematic review and meta-analysis to clarify the role of vitamin D in patients with NAFLD.

## 2. Materials and Methods

### 2.1. Search strategy

**Figure 1.**
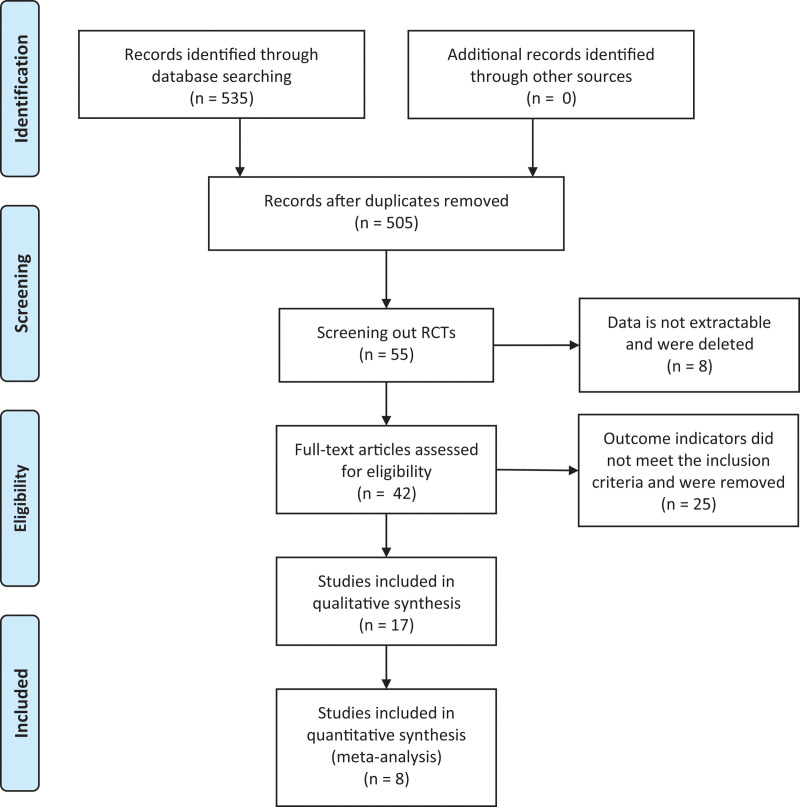
Literature screening process.

National Library of Medicine, Cochrane Library, China National Knowledge Infrastructure, Web of Science, WANFANG databases, and Google Scholar were retrieved to collect relevant randomized controlled trials (RCTs), which are published from the earliest records to April 2023. The search terms are “vitamin D,” “nonalcoholic fatty liver disease,” “vitamin D3,” “cholecalciferol,” “ergocalciferol,” “NAFLD” in combination. The literature screening process is shown in Figure 1.

### 2.2. Inclusion criteria

(1) NAFLD or nonalcoholic steatohepatitis aged > 18 years; (2) placebo or lifestyle intervention in the control group, and oral or injectable vitamin D or D3 in the vitamin D group; (3) outcome indicators include the levels of 25(OH)D, homeostasis model assessment of insulin resistance (HOMA-IR), fasting blood glucose (FBG), and fasting insulin (FINS); (4) study design: RCTs.

### 2.3. Exclusion criteria

(1) Inaccessible abstracts or full text; (2) studies for which data could not be accurately extracted; (3) duplicate published studies.

### 2.4. Literature quality assessment

The evaluator first independently completes an initial screening of the included literature by reading the title and abstract. After reading the full text, literatures met the inclusion criteria were included. The following evaluation criteria for RCTs in Review Manager 5.4 were used: (1) generation of random sequences; (2) allocation concealment; (3) blinding of subjects and intervention providers; (4) blinding of outcome assessments; (5) completeness of outcome data; (6) selective outcome reporting; and (7) other biases. The 7 items above were evaluated using “low risk,” “high risk,” and “uncertain risk.”

### 2.5. Data extraction

Data extracted from the included literatures included the following: (1) general information such as title, first author, year of publication, sample size and trial quality score; (2) comparability of data and interventions across patient data groups; and (3) outcome indicators: levels of 25(OH)D, HOMA-IR, FBG and FINS.

### 2.6. Statistical processing

Data were analyzed using Review Manager 5.4 software at a test level of α = 0.05. Continuous variables and units were analyzed by mean difference (MD) and its 95% confidence intervals (CI). Clinical heterogeneity of included studies was first analyzed, followed by statistical heterogeneity using the *I*^2^ test.^[9]^ When *P* > .1, *I*^2^ < 50%, homogeneity among several similar studies could be considered and a fixed-effects model was chosen for meta-analysis. Heterogeneity was considered when *P* < .1, *I*^2^ > 50%; and a random-effect model was used. *P* < .05 indicates a statistically significant difference and *I*^2^ ≥ 50% suggests high heterogeneity.^[10]^

## 3. Results

### 3.1. Study characteristics

**Figure 2.**
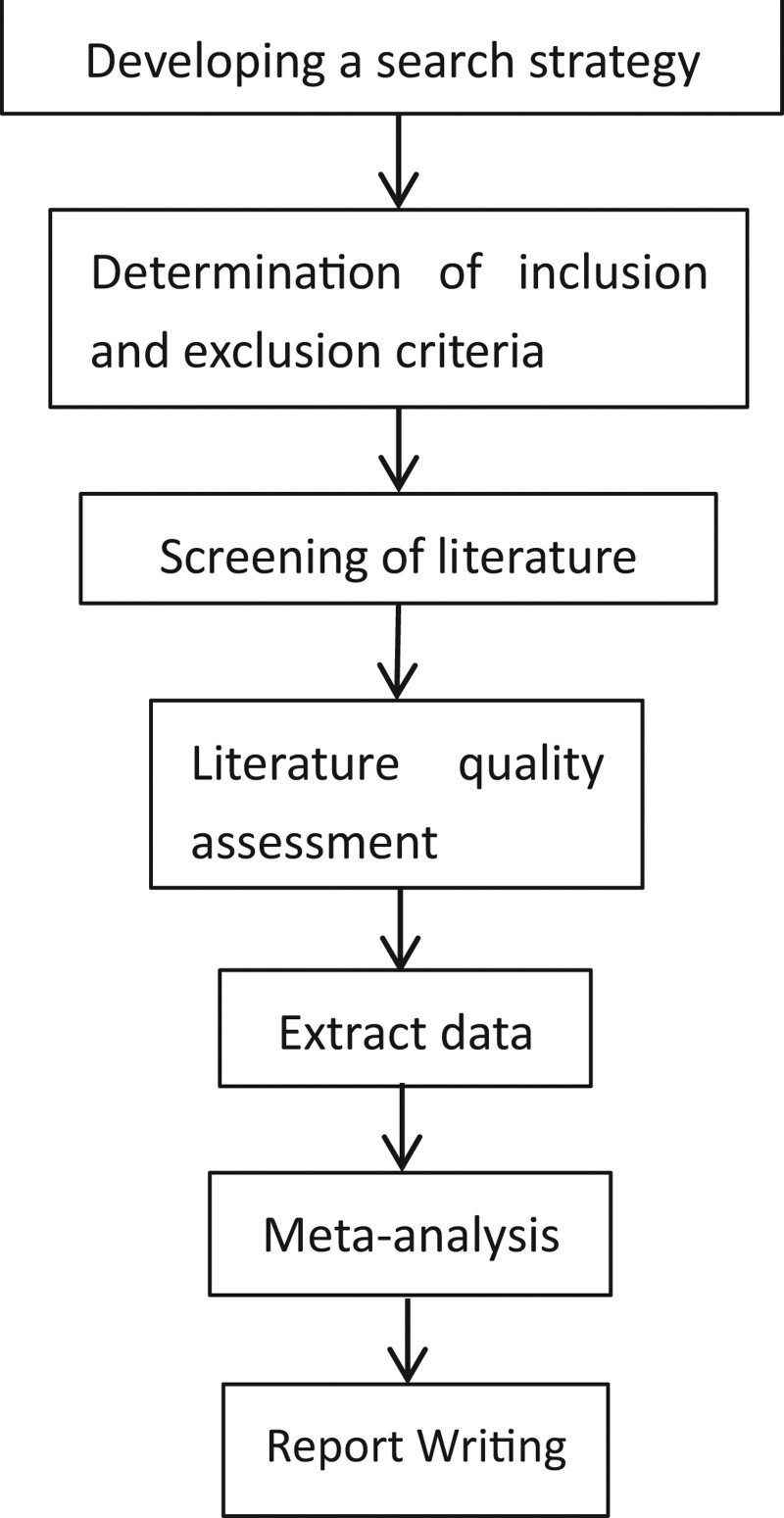
Flow chart.

Figure 2 is the flow chart. The initial review obtained 535 papers, and finally 8 RCTs are included.^[11–18]^ There are 657 patients totally. 329 patients are in the control group and 328 patients are in the experimental group. The basic characteristics of includes studies are shown in Table 1.

**Table 1:** Basic information of inclusion in the study.

Author	Number of patients	Vitamin D supplementation dose	Periodicity	Cross referencing measures	Study outcome indicators
Hamid L (2016)	T:40 C:40	25 μg/d	12 week	Placebo	①②③④
Hamid Y (2021)	T:64 C:64	0.25 μg/d	17 week	Placebo	①②③
Mahdi (2014)	T:30 C:30	5000 U/w	10 week	Placebo	④
Mazhar (2019)	T:51 C:51	5000 U/w	12 week	Placebo	①④
Dai Jiale (2022)	T:50 C:50	400 U/d	12 week	Lifestyle interventions	①②③④
Han Yuanping (2022)	T:14 C:15	Single intramuscular injection of vitamin Vitamin D3 600,000 U	1 month	No intervention	③
Li Jinqiang (2022)	T:42 C:42	0.25 μg/d	12 week	Lifestyle interventions	④
Lu Yongwen (2019)	T:37 C:37	0.5 μg/d	12 week	Lifestyle interventions	①②③④

① Insulin resistance index. ② Fasting blood glucose. ③ Insulin level. ④ 25(OH)D.

C = control group, T = test group.

### 3.2. Quality assessment

All 8 studies are low risk according to the randomized allocation. Seven studies are low risk and one study is unclear in the allocation concealment. Three studies are low risk and 5 studies are unclear in the double-blind method. Two studies are low risk and 6 studies are unclear in the evaluation of blindness. Four studies are low risk and 4 studies are unclear in the data integrity. Five studies are low risk and 3 studies are unclear in the selective report. Six studies are low risk and 2 studies are unclear in the others. Since there is no high risk in these 7 criteria, the quality of all included studies is good. The quality assessment table is shown in Table 2.

**Table 2:** Quality assessment table.

Included studies	Random allocation	Allocation concealment	Double blind method	Evaluation of blindness	Data integrity	Selective report	Others
Hamid L (2016)	Low risk	Low risk	Unclear	Unclear	Low risk	Unclear	Low risk
Hamid Y (2021)	Low risk	Low risk	Low risk	Low risk	Unclear	Low risk	Low risk
Mahdi (2014)	Low risk	Low risk	Low risk	Low risk	Low risk	Unclear	Low risk
Mazhar (2019)	Low risk	Low risk	Low risk	Unclear	Unclear	Low risk	Unclear
Dai Jiale (2022)	Low risk	Unclear	Unclear	Unclear	Low risk	Low risk	Unclear
Han Yuanping (2022)	Low risk	Low risk	Unclear	Unclear	Low risk	Unclear	Low risk
Li Jinqiang (2022)	Low risk	Low risk	Unclear	Unclear	Unclear	Low risk	Low risk
Lu Yongwen (2019)	Low risk	Low risk	Unclear	Unclear	Unclear	Low risk	Low risk

### 3.3. Meta-analysis results

#### 3.3.1. 25(OH)D.

**Figure 3.**
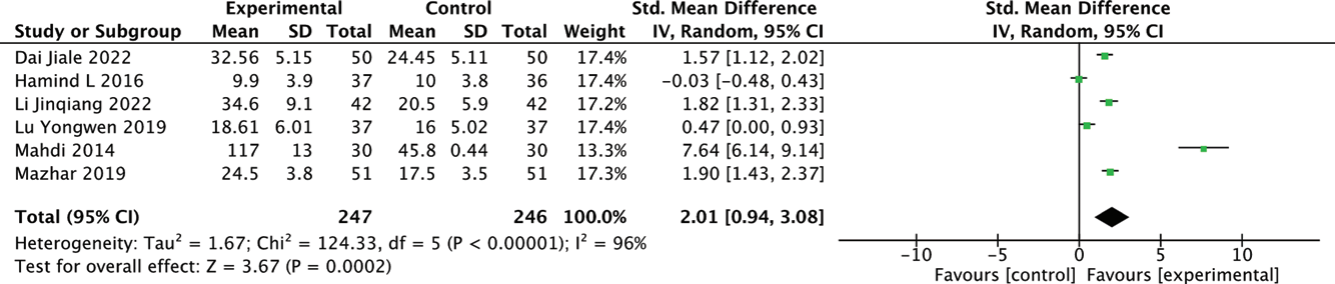
Changes of 25(OH)D between 2 groups. 25(OH)D = 25-hydroxyvitamin D.

**Figure 4.**
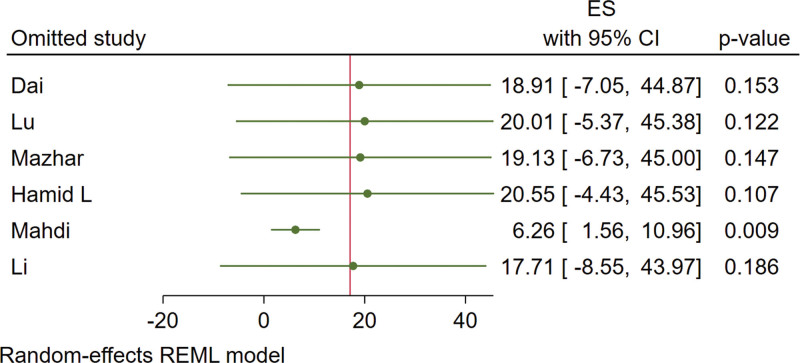
Effect sizes of 25(OH)D under leave-one-out approach. 25(OH)D = 25-hydroxyvitamin D.

Six studies report changes in 25(OH)D in patients. The results are shown in Figure 3. There is a heterogeneity as *I*^2^ = 96% and a random-effect model was used. Under a leave-one-out approach, the heterogeneity is possibly caused by the research of Mahdi (Fig. 4). Vitamin D supplementation significantly increased serum 25(OH)D levels (MD = 2.01, 95% CI: 0.94 to 3.08, *P* < .05).

#### 3.3.2. HOMA-IR.

**Figure 5.**
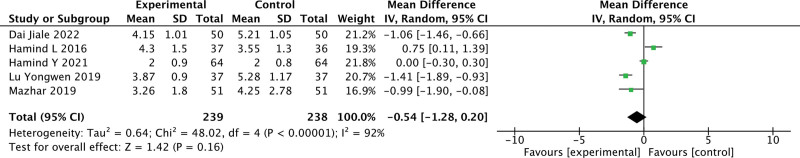
Changes of HOMA-IR between 2 groups. HOMA-IR = homeostasis model assessment of insulin resistance.

**Figure 6.**
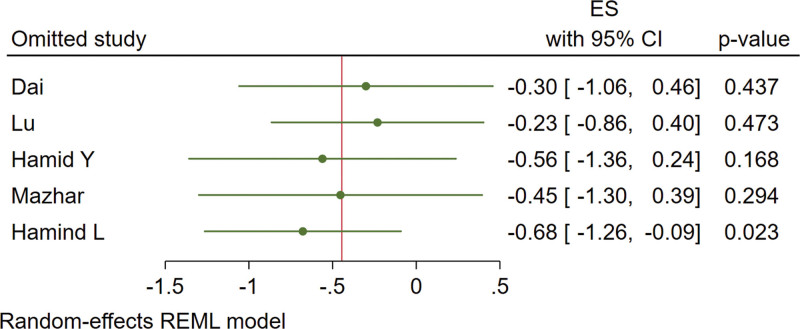
Effect sizes of HOMA-IR under leave-one-out approach. HOMA-IR = homeostasis model assessment of insulin resistance.

Five studies are included. The results are shown in Figure 5. There is a heterogeneity as *I*^2^ = 92% and a random-effect model was used. Under a leave-one-out approach, the heterogeneity is possibly caused by the research of Hamind L and Hamind Y (Fig. 6). There is no statistically significant difference in the effect of vitamin D supplementation on HOMA-IR (MD = -0.54, 95% CI: -1.28 to 0.20, *P* = .16).

#### 3.3.3. FBG.

**Figure 7.**
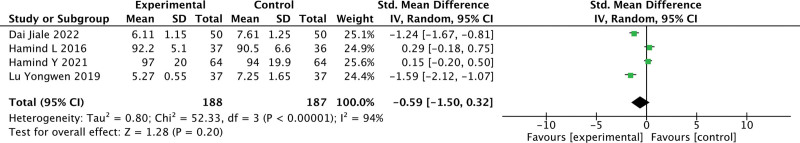
Changes of FBG between 2 groups. FBG = fasting blood glucose.

**Figure 8.**
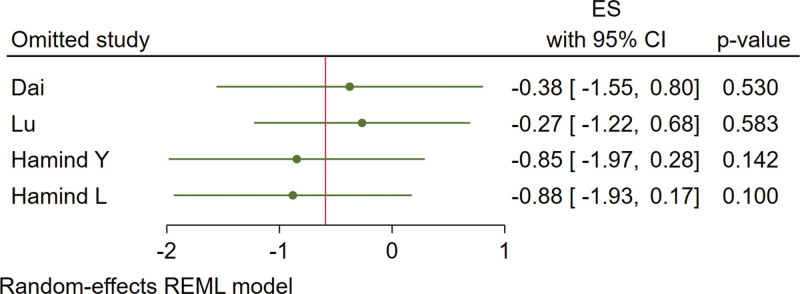
Effect sizes of FBG under leave-one-out approach. FBG = fasting blood glucose.

Four studies are included. The results are shown in Figure 7. There is a heterogeneity as *I*^2^ = 94% and a random-effect model was used. Under a leave-one-out approach, the heterogeneity is possibly caused by the research of Hamind L and Hamind Y (Fig. 8). There is no statistically significant difference in the effect of vitamin D supplementation on FBG (MD = −0.59, 95% CI: −1.50 to 0.32, *P* = .20).

#### 3.3.4. FINS.

**Figure 9.**
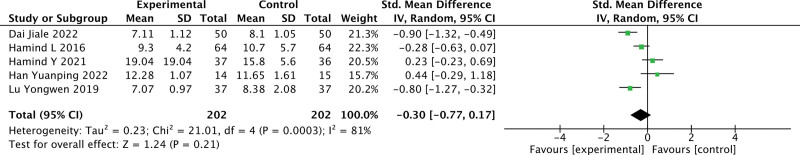
Changes of FINS between 2 groups. FINS = fasting insulin levels.

**Figure 10.**
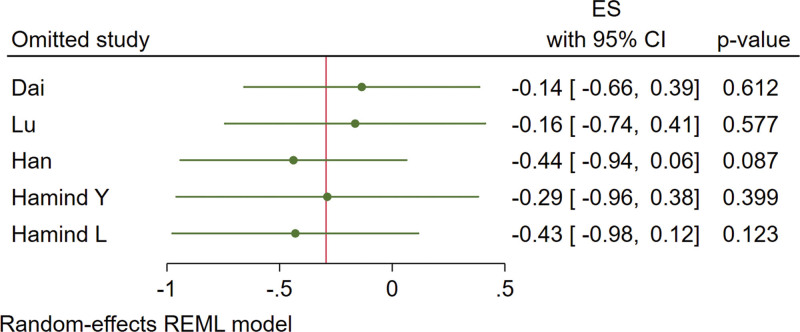
Effect sizes of FINS under leave-one-out approach. FINS = fasting insulin levels.

Five studies are included. The results are shown in Figure 9. There is a heterogeneity as *I*^2^ = 81% and a random-effect model was used. Under a leave-one-out approach, the heterogeneity is possibly caused by the research of Hamind L and Han (Fig. 10). There is no statistically significant difference in the effect of vitamin D supplementation on FINS (MD = −0.30, 95% CI: −0.77 to 0.17, *P* = .21).

#### 3.3.5. Publication bias.

**Figure 11.**
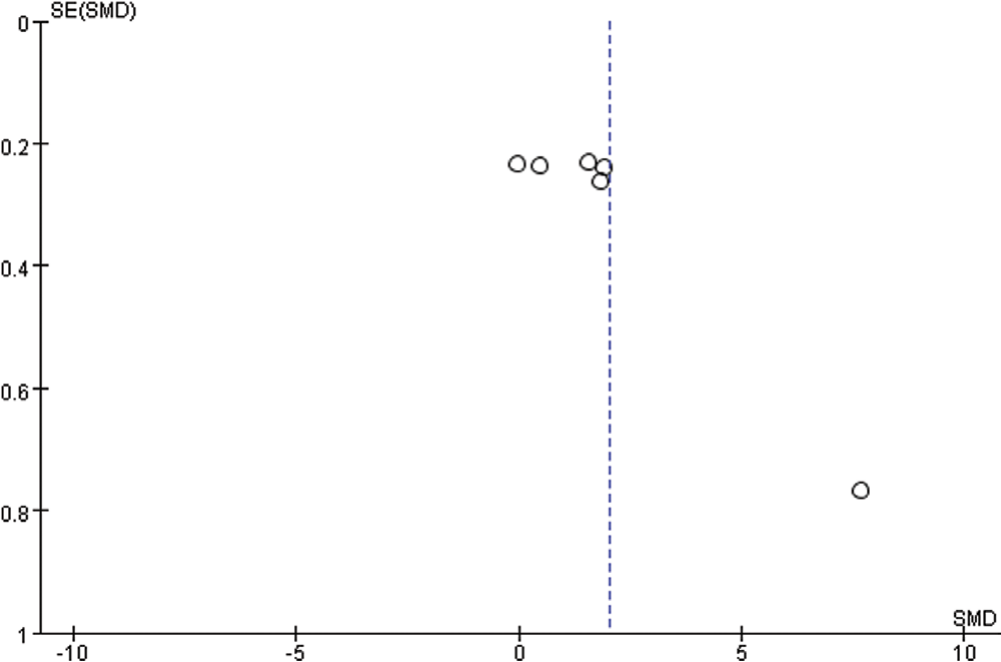
Funnel plot.

As shown in Figure 11, based on the 25(OH)D level, funnel plot is applied to evaluate the publication biases of 6 studies. It shows that the publication bias is small.

## 4. Discussion

In this meta-analysis, vitamin D supplementation increased 25(OH)D levels significantly. However, there is no significant effect on HOMA-IR, FBG, or FINS. Also, heterogeneity exists in all indicators. In the included studies, patients in both groups have only placebo and lifestyle interventions without taking statins or any other medicines. The reason for no treatment of statins in related RCTs is probably that statins have no influence on plasma vitamin D levels.^[19]^

Since 25(OH)D has a half-life of approximately 3 weeks, heterogeneity within the HOMA-IR group disappears and FINS reduces when the duration of administration is prolonged (>16 weeks).^[4]^ It suggests that vitamin D supplementation in improving IR is based on a low baseline level of vitamin D, so vitamin D sufficiency may lead to a relative insensitivity to vitamin D supplementation.^[20]^ In addition, a recent RCT suggests that vitamin D supplementation may not improve glycemic index or anthropometry in patients with NAFLD.^[21]^ It found that 25(OH) D levels are lower in the NAFLD group than in the control group, thus suggesting a negative correlation between 25(OH) D levels and NAFLD.^[1]^ A meta-analysis shows that lower serum 25(OH)D concentrations (mainly in patients with vitamin D deficiency) are the only significant predictor of NAFLD.^[22]^ These are generally consistent with the findings in this meta-analysis.

This meta-analysis focuses on assessing indexes related to glucose metabolism but not liver enzymes or lipids. Active vitamin D is excluded from this study, since supplementation of active vitamin D cannot increase circulating levels of 25(OH)D. In extrarenal tissues, free 25(OH)D metabolized by 1-alpha-hydroxylase to 1,25(OH)2D, then it binds to the vitamin D receptor to regulate gene transcription and perform physiological functions.^[23]^

This study has some limitations: (1) the number of populations included in the study is relatively small; (2) there is heterogeneity, which may be related to duration of dosing, route of administration and the differences within baseline levels of 25(OH)D.

## 5. Conclusion

Vitamin D supplementation improves 25(OH)D levels in patients with NAFLD, but there is no effect on HOMA-IR, FBG, or FINS.

## Acknowledgments

The authors thank Dr Bin Wang for assistance with data extraction.

## Author contributions

**Conceptualization:** Xuemeng Chen, Ye Zhao, Yan Zhao.

**Funding acquisition:** Ye Zhao, Liheng Dai.

**Investigation:** Xuemeng Chen.

**Methodology:** Liheng Dai.

**Project administration:** Ran Zhang.

**Resources:** Ye Zhao, Yan Zhao.

**Supervision:** Liheng Dai.

**Validation:** Yan Zhao, Liheng Dai.

**Writing – original draft:** Xuemeng Chen, Ye Zhao.

**Writing – review & editing:** Ran Zhang, Yan Zhao, Liheng Dai.
